# A Case of Markedly Elevated Isolated Alkaline Phosphatase in the Third Trimester of Pregnancy

**DOI:** 10.1155/2022/1611304

**Published:** 2022-04-27

**Authors:** Courtney T. Connolly, Olivia Grubman, Zainab Al-Ibraheemi, Tatyana Kushner

**Affiliations:** ^1^Department of Obstetrics, Gynecology and Reproductive Science, Icahn School of Medicine at Mount Sinai, New York City, USA; ^2^Department of Medicine and Liver Disease, Icahn School of Medicine at Mount Sinai, New York City, USA

## Abstract

**Background:**

Alkaline phosphatase (ALP) is an enzyme produced by the liver, small intestine, bone, and kidneys as well as the placenta during pregnancy. ALP levels may increase up to twice the normal limit during pregnancy secondary to placental release and fetal bone growth. Rare case reports of extremely elevated levels of ALP during pregnancy have demonstrated possible association with adverse pregnancy outcomes.

**Case:**

The patient is a 36-year-old G2P1001 who was found to have extremely elevated ALP levels during pregnancy after presenting with bilateral lower leg swelling and rash after receiving the Pfizer COVID-19 vaccine. She subsequently developed intrahepatic cholestasis of pregnancy and preeclampsia. ALP peaked at 2,601 U/L immediately prior to delivery at 36 weeks 1 day. She was followed postpartum, and her ALP levels had nearly normalized by 15 weeks postpartum.

**Conclusion:**

Our case demonstrates a rare report of an extremely elevated level of ALP in the setting of multiple adverse pregnancy outcomes, including preterm delivery, preeclampsia without severe features, and intrahepatic cholestasis of pregnancy.

## 1. Introduction

Alkaline phosphatase (ALP) is an enzyme produced by various tissues, including the liver, small intestine, bones, kidneys, and placenta during pregnancy. It is not uncommon to see elevations in ALP during pregnancy, and by the 15^th^ to 26^th^ weeks of pregnancy, term placental ALP isozymes can be detected in the maternal serum [1]. These isozymes are likely produced by syncytiotrophoblasts in the placenta and are theorized to play a role in transport across cell membranes and metabolism [1, 2]. ALP increases with increasing gestational age, with contributions likely from both placental isoenzymes. ALP peaks in the third trimester and usually reaches levels double that of a nonpregnant individual but normalizes postpartum [2–5]. However, it is important to exclude other causes of elevated ALP out of proportion to what is expected when detected during pregnancy, including malignancy, drugs, and bone, renal, and hepatic diseases.

Few cases of extremely elevated levels of ALP have been reported in the literature, and ALP has been associated with adverse outcomes including low birth weight [6, 7], intrauterine growth restriction [8], preterm delivery [9, 10], and hypertensive disorders of pregnancy [11]. It has been thought that ALP may be a marker of uteroplacental vascular disease [12].

We describe a case of extremely elevated ALP detected during a pregnancy complicated by preterm delivery, intrahepatic cholestasis of pregnancy, and preeclampsia. There were no adverse fetal effects. ALP levels returned to near baseline by 15 weeks postpartum.

## 2. Case Presentation

A 36-year-old gravida 2 para 1001 at 29 weeks 6 days, dated by a 14-week ultrasound inconsistent with her last menstrual period, re-presented to the high-risk obstetrics clinic where she was previously followed for type 1 diabetes for evaluation of a new finding of abnormally elevated alkaline phosphatase. She had been seen in the Emergency Department (ED) two days prior where she reported the development of bilateral ankle rash, pain, leg swelling, and subjective fever 1 day after receiving the Pfizer COVID-19 vaccine. She denied chills, chest pain, dyspnea, myalgias, abdominal pain, and exposure to irritating substances or new clothing in the area of her rash. There were no symptoms, focal signs of infection, or recent travel to suggest exposure to the Zika virus. Her medical history was significant for bipolar disorder, prior cholecystectomy, hypothyroidism stable on levothyroxine 50 mcg daily, and type 1 diabetes mellitus complicated by suboptimal glucose control in the current pregnancy and two prior hospital admissions for diabetic ketoacidosis, most recently 4 years ago.

On presentation to the ED, she was afebrile, and vital signs were stable. Physical exam revealed 1+ bilateral pitting edema and a nonblanching confluent erythematous rash on the medial and posterior of the lower extremities. Abdominal exam was unremarkable aside from gravid uterus, and the rash was not warm to the touch.

Bilateral lower extremity Doppler ultrasound was negative for deep venous thrombosis. Basic labs were ordered as part of her work-up, and a comprehensive metabolic panel was notable for alkaline phosphatase (ALP) of 1,327 U/L and albumin of 2.5 g/dL. AST, ALT, total protein, and total and direct bilirubin were within normal limits. There was no leukocytosis, anemia, or thrombocytopenia. Her last ALP measured at 16 weeks 4 days was 258, demonstrating significant interval change.

Given her benign abdominal exam and labs not suggestive of an active infection, she was discharged with close outpatient follow-up. She was seen two days later in the obstetrics high-risk clinic, where she was noted to be clinically stable. She was then referred to the hepatology practice for further evaluation and was seen at 31 weeks 6 days. On further history, she reported no use of acetaminophen or nonsteroidal anti-inflammatory drugs, alcohol, intravenous drug, herbal supplements, and antibiotics and denied recent travel. Family history was positive for Hepatitis C infection in her brother, most likely acquired secondary to intravenous drug use. She was immune to Hepatitis B. She noted pruritus and continued to endorse bilateral pedal edema with improvement in the rash.

Further work-up at that time was significant for ALP now elevated to 1,937 U/L, albumin of 2.3 g/dL, total bile acids elevated at 22 *μ*mol/L, and ceruloplasmin of 63.4 mg/dL. Mitochondrial (M2) antibody, Hepatitis A IgM, and Hepatitis E IgM were negative. GGT was normal. Complete blood count with differential, ALT, AST, total and direct bilirubin, and serum alpha-1-antitrypsin were within normal limits. Hepatitis C RNA was not detected. Liver ultrasound showed normal size at 16.0 cm, normal contour, mildly increased echogenicity, extrahepatic common bile duct 0.2 cm, and a surgically absent gallbladder, compatible with nonspecific hepatic parenchymal disease.

She was diagnosed with intrahepatic cholestasis of pregnancy with plan for induction of labor at 37 weeks and was started on ursodiol 300 mg TID. She was evaluated again at 34 weeks 6 days by the high-risk obstetrics and liver medicine teams, reporting continued pruritus refractory to topical treatments and ursodiol. Follow-up labs revealed bile acids of 35 *μ*mol/L, albumin of 2.2 g/dL, and ALP of 2,069 IU/L. Alkaline phosphatase isoenzymes were liver fraction of 59%, bone fraction of 40%, and intestinal fraction of 1%. Placental fraction was not able to be quantified by the specific laboratory processing the specimen.

She presented to Labor and Delivery triage at 35 weeks 2 days with mild range blood pressures, elevated urine protein/creatinine ratio of 824 mgTP/gCr, and otherwise normal labs, which was consistent with the diagnosis of preeclampsia without severe features. She re-presented at 35 weeks 4 days in latent labor. She delivered a liveborn female weighing 3,020 grams via normal spontaneous vaginal delivery, and Apgar scores were 8 and 8. Placental pathology demonstrated a 625-gram placenta with mild intervillous fibrin deposition. On pathology, the fetal membranes had mild focal acute chronic inflammation. No special stains were performed.

**Figure 1:**
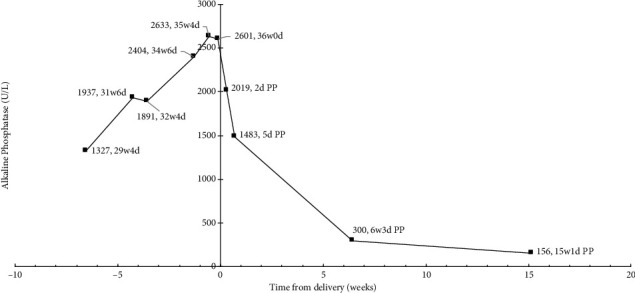
Total alkaline phosphatase levels over time relative to delivery at 36 weeks 1 day. PP: postpartum.

Her ALP peaked at 2,601 U/L (approximately 22 times the upper limit of normal) (ALP trend demonstrated in Figure 1), the final measurement before delivery at 35 weeks 5 days. By postpartum day 2, ALP was 2,019 U/L, and she was discharged in stable condition. She was seen again on postpartum day five for preeclampsia postpartum monitoring, and ALP had decreased to 1,483 U/L. At her six-week postpartum visit, ALP was 300 U/L, and by 15 weeks postpartum, it was 156 U/L.

## 3. Discussion

Our case report is one of few in the literature that describes an extremely elevated level of ALP discovered incidentally during pregnancy that was later complicated by intrahepatic cholestasis of pregnancy, preterm delivery, and preeclampsia. In our case, the patient delivered at 36 weeks 1 day, and alkaline phosphatase levels had nearly normalized by 15 weeks postpartum.

A review of previous case reports demonstrates an association between elevated ALP and adverse pregnancy outcomes. Specifically, several patient cases documented an association with mild hypertension or other hypertensive disorder of pregnancy [2, 13, 14]. A case control study by Rajagambeeram et al. further supported this finding and showed that total serum ALP and placental (heat stable) ALP were significantly elevated in women with hypertensive disorders of pregnancy versus controls [11]. Other cases reported associations with preterm delivery [8, 14], preterm premature rupture of membranes [15], gestational diabetes [14, 16, 17], and intrauterine growth restriction [8]. One case report interestingly noted thrombocytopenia in the mother with extremely elevated placental isoenzymes, and upon further investigation was found to have several relatives with elevated baseline ALP suggesting a possible genetic link [18]. In addition, while the majority of cases of this extreme elevation in ALP have been associated with adverse pregnancy outcomes or pregnancy complications, it is important to note that there were also several case reports of elevated ALP in an otherwise uncomplicated pregnancy [19, 20].

Our case aligns with several of the associations suggested by previous reports, specifically preterm delivery and hypertensive disorder of pregnancy. However, our case was unique in that we report an association that developed after administration of a vaccination and the presence of elevated bile acids suggesting a diagnosis of intrahepatic cholestasis of pregnancy. While a rise in ALP may occur with intrahepatic cholestasis of pregnancy, an increase of 22x the upper limit of normal range is unusually high. Interestingly, *in vitro fertilization* has also been found to be associated with cholestasis of pregnancy, but there is no current evidence suggesting that extreme elevations in alkaline phosphatase are associated with assisted reproductive technology.

The vast majority of reported cases, if able to speciate the isoenzymes, found placental isoenzyme to be the predominant fraction [2, 13–16, 18–20]. There was a rare case of elevation in the bone isoenzyme in a patient who was discovered to have an elevated ALP of 1525 U/L at the onset of labor induction despite high-normal ALP earlier in pregnancy with rapid progression to 2561 U/L 95 minutes later [17].

Most cases of elevated alkaline phosphatase resolved within six to 12 weeks postpartum [2, 8, 15–20]. However, one case took up to 18 weeks to resolve, which appears to be more consistent with the timeline of our patient's course [13] (Figure 1). This may be partially explained by the relatively longer half-life of placental isoenzymes of ALP (approximately 7 days) versus liver and bone isoenzymes (1-3 days) [19].

Finally, it is important to emphasize that other causes of elevated ALP must be excluded. Drug-induced liver injury secondary to the COVID-19 vaccination was considered in our patient's case. However, the onset of lab abnormalities in temporal relation to the vaccination does not suggest an immunologic reaction, and this is not an established adverse effect of the COVID-19 vaccine. In cases of elevated ALP levels, it should be determined early on whether it is of isolated hepatic origin, bone, or placental origin. A concurrent elevation in GGT likely indicates that the ALP is hepatic in origin, and appropriate work-up with further labs and ultrasound imaging should be performed to investigate for intrahepatic causes (e.g., primary biliary cholangitis, primary sclerosing cholangitis (PSC), viral hepatitis, and sarcoidosis) and extrahepatic causes (e.g., PSC, bile duct strictures, and stones). An elevated bone fraction of ALP indicates increased bone turnover, which can be attributed to diseases such as osteosarcoma and metastatic cancer as well as hyperparathyroidism, Paget disease, osteomalacia, and others. Our patient had a normal gamma-glutamyl transpeptidase as well as normal liver, bone, and intestinal fractions of ALP but an overall elevation in total ALP. While we could not fractionate the placental isoenzyme, we presume that it is possible the placental isoenzyme may have been elevated in our patient's case. Of note, the placental ALP isoenzyme is heat labile and migrates with the bone fraction. For future cases, we suggest sending these samples to a laboratory that is capable of processing all isoenzyme types and specifically quantifying the placental isozyme. There is a Regan isoenzyme that is a placental-type isoenzyme and has been associated with ovarian serous cystadenocarcinoma, lung cancer, and seminoma [13]. Our team requested placental pathology results to investigate whether there were any placental factors that contributed to such a high release of alkaline phosphatase. We believe there may be an association between placental pathology, preeclampsia, and alkaline phosphatase. However, we cannot prove this with our case report and suggest further studies to investigate.

In conclusion, we report a case of incidentally diagnosed markedly elevated ALP in a pregnancy complicated by intrahepatic cholestasis of pregnancy, preterm delivery, and preeclampsia without severe features. Our case report adds to the growing body of literature that suggests elevated ALP levels may be a harbinger of adverse pregnancy outcomes and highlights the importance of fractionating the isoenzymes and continuing close follow-up during pregnancy and postpartum. However, future large studies are needed to establish ALP as a marker of a placental pathology or high-risk pregnancy.
